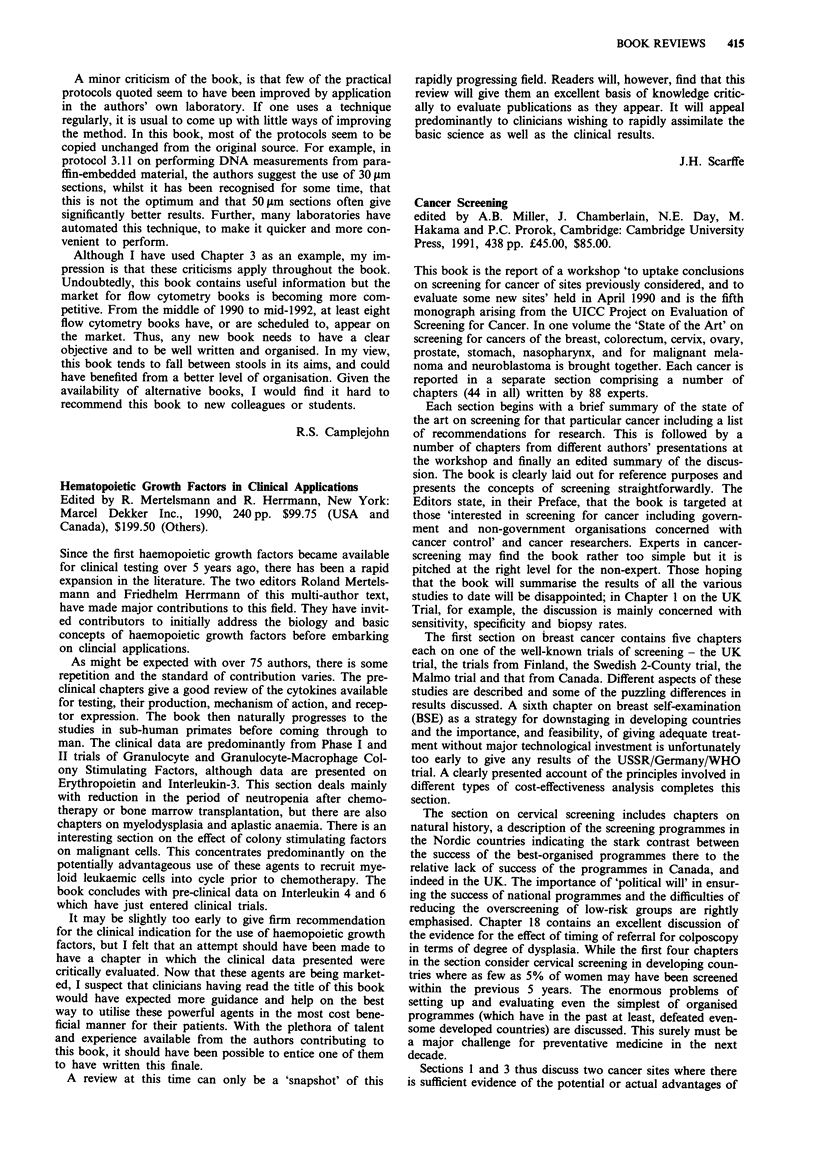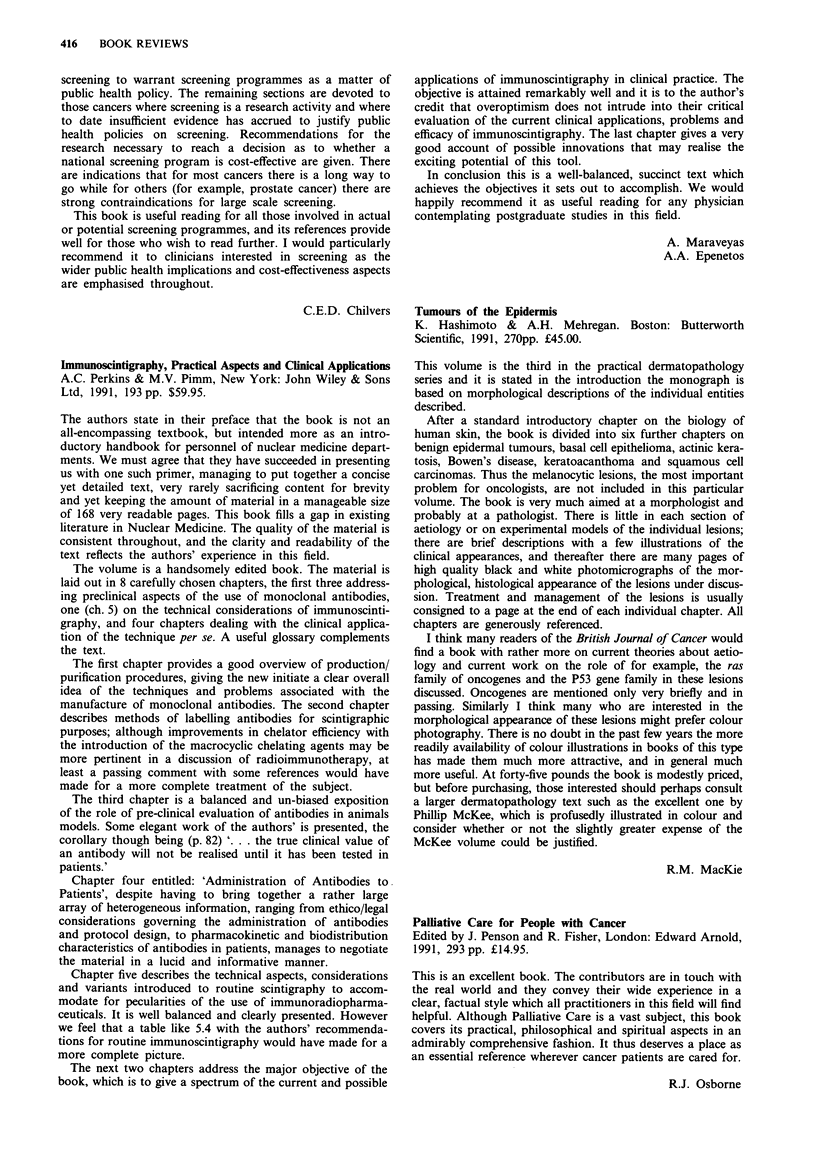# Cancer Screening

**Published:** 1992-08

**Authors:** C.E.D. Chilvers


**Cancer Screening**
edited by A.B. Miller, J. Chamberlain, N.E. Day, M. Hakama and P.C. Prorok, Cambridge: Cambridge University Press, 1991, 438 pp. £45.00, $85.00.

This book is the report of a workshop 'to uptake conclusions on screening for cancer of sites previously considered, and to evaluate some new sites' held in April 1990 and is the fifth monograph arising from the UICC Project on Evaluation of Screening for Cancer. In one volume the 'State of the Art' on screening for cancers of the breast, colorectum, cervix, ovary, prostate, stomach, nasopharynx, and for malignant melanoma and neuroblastoma is brought together. Each cancer is reported in a separate section comprising a number of chapters (44 in all) written by 88 experts.

Each section begins with a brief summary of the state of the art on screening for that particular cancer including a list of recommendations for research. This is followed by a number of chapters from different authors' presentations at the workshop and finally an edited summary of the discussion. The book is clearly laid out for reference purposes and presents the concepts of screening straightforwardly. The Editors state, in their Preface, that the book is targeted at those 'interested in screening for cancer including government and non-government organisations concerned with cancer control' and cancer researchers. Experts in cancer-screening may find the book rather too simple but it is pitched at the right level for the non-expert. Those hoping that the book will summarise the results of all the various studies to date will be disappointed; in Chapter 1 on the UK Trial, for example, the discussion is mainly concerned with sensitivity, specificity and biopsy rates.

The first section on breast cancer contains five chapters each on one of the well-known trials of screening – the UK trial, the trials from Finland, the Swedish 2-County trial, the Malmo trial and that from Canada. Different aspects of these studies are described and some of the puzzling differences in results discussed. A sixth chapter on breast self-examination (BSE) as a strategy for downstaging in developing countries and the importance, and feasibility, of giving adequate treatment without major technological investment is unfortunately too early to give any results of the USSR/Germany/WHO trial. A clearly presented account of the principles involved in different types of cost-effectiveness analysis completes this section.

The section on cervical screening includes chapters on natural history, a description of the screening programmes in the Nordic countries indicating the stark contrast between the success of the best-organised programmes there to the relative lack of success of the programmes in Canada, and indeed in the UK. The importance of 'political will' in ensuring the success of national programmes and the difficulties of reducing the overscreening of low-risk groups are rightly emphasised. Chapter 18 contains an excellent discussion of the evidence for the effect of timing of referral for colposcopy in terms of degree of dysplasia. While the first four chapters in the section consider cervical screening in developing countries where as few as 5% of women may have been screened within the previous 5 years. The enormous problems of setting up and evaluating even the simplest of organised programmes (which have in the past at least, defeated even-some developed countries) are discussed. This surely must be a major challenge for preventative medicine in the next decade.

Sections 1 and 3 thus discuss two cancer sites where there is sufficient evidence of the potential or actual advantages of screening to warrant screening programmes as a matter of public health policy. The remaining sections are devoted to those cancers where screening is a research activity and where to date insufficient evidence has accrued to justify public health policies on screening. Recommendations for the research necessary to reach a decision as to whether a national screening program is cost-effective are given. There are indications that for most cancers there is a long way to go while for others (for example, prostate cancer) there are strong contraindications for large scale screening.

This book is useful reading for all those involved in actual or potential screening programmes, and its references provide well for those who wish to read further. I would particularly recommend it to clinicians interested in screening as the wider public health implications and cost-effectiveness aspects are emphasised throughout.

C.E.D. Chilvers